# Supplementary material for: Finger Kinematics during Human Hand Grip and Release
Source: Biomimetics (Basel). 2023 Jun 8;8(2):244. doi: 10.3390/biomimetics8020244 (PMC10296280; doi:10.3390/biomimetics8020244)
Supplement: Supplementary file 1 [file biomimetics-08-00244-s001.zip › biomimetics-2381688-supplementary.pdf]

Supplementary Materials:

The angular values of finger joints at maximum flexion and extension for all the participants are shown in Table S1 and Table S2, which are used to calculate the dynamic ROMs. The peak velocities of finger joints during flexion and extension for all the participants are shown in Table S3 and Table S4. The detailed information on feature points of joints at different motion phases is shown in Table S5, Table S6, Table S7, Table S8, Table S9 and Table S10, which is used to determine the joint sequence. The detailed information on feature points of fingers at different motion phases is shown in Table S11 and Table S12, which is used to determine the finger sequence.

Table S1. The angle of finger joints at maximum flexion during grasping and releasing for 22 subjects.

Angle (°)	Thumb		Index			Middle			Ring			Little		
	IP	MCP	DIP	PIP	MCP	DIP	PIP	MCP	DIP	PIP	MCP	DIP	PIP	MCP
S1	75.33	39.92	61.37	101.43	86.76	70.85	97.65	82.24	76.54	98.65	80.87	70.12	86.66	77.27
S2	50.18	35.25	66.13	92.48	74.36	75.32	94.60	78.39	72.08	92.00	85.23	76.27	92.55	83.14
S3	69.31	57.28	62.18	96.89	85.11	78.49	89.28	84.11	85.45	92.96	87.61	69.36	91.39	87.49
S4	62.50	46.89	70.73	102.7	88.74	67.61	96.64	84.03	74.03	102.30	96.48	74.98	90.55	87.53
S5	67.30	42.51	67.63	99.25	93.00	81.14	104.7	91.75	78.12	100.86	90.39	74.86	93.49	80.51
S6	64.71	49.50	66.05	87.85	72.60	76.32	97.42	83.46	73.61	96.35	82.10	64.51	86.71	80.19
S7	56.06	34.27	60.88	93.17	79.93	72.48	93.15	86.65	68.89	103.93	82.77	75.19	90.57	82.06
S8	57.71	35.46	72.89	92.10	84.95	69.71	91.77	89.44	77.96	96.46	93.81	75.38	91.69	82.60
S9	78.30	47.18	67.06	102.3	92.45	80.95	94.73	85.49	70.54	90.07	80.79	69.35	86.06	83.45
S10	64.95	40.00	65.36	92.30	86.74	73.39	87.63	83.19	71.14	88.00	82.49	66.79	85.44	80.55
S11	65.09	47.19	74.51	96.11	86.38	75.35	96.09	88.38	75.53	95.30	87.40	73.06	87.85	82.62
S12	72.86	51.88	54.03	93.53	83.42	79.94	93.51	90.21	70.49	98.83	90.48	75.67	95.59	85.76
S13	59.14	36.38	65.18	98.79	87.79	80.79	103.92	84.16	72.25	97.83	80.63	75.52	95.31	82.27
S14	69.52	44.84	69.47	100.98	82.47	70.23	95.23	88.13	62.33	98.99	84.31	64.49	84.36	78.19
S15	70.60	38.40	64.87	99.05	83.79	68.67	96.35	89.86	80.47	92.06	85.48	73.02	90.72	79.81
S16	71.59	43.16	77.27	102.53	86.51	72.89	90.46	87.69	67.09	95.96	83.89	68.47	92.65	83.44
S17	57.41	47.12	69.24	99.43	85.69	68.10	92.22	86.44	75.03	92.39	90.69	70.36	96.61	88.38
S18	78.72	55.57	70.62	99.97	83.34	80.79	96.39	82.37	74.52	101.54	82.82	74.60	90.28	83.24
S19	73.04	57.32	66.54	96.44	85.97	75.07	97.53	84.62	77.27	95.68	81.00	77.86	93.10	83.88
S20	71.28	46.72	65.46	95.67	82.48	70.36	97.48	82.81	66.09	91.28	83.69	65.09	90.22	85.23
S21	71.75	47.54	66.43	97.07	80.36	73.91	91.54	80.25	72.64	94.07	84.27	70.98	89.75	79.63
S22	74.02	41.14	73.55	100.12	87.77	71.91	91.04	82.45	84.78	92.04	86.51	76.34	88.80	85.96
Mean	67.33	44.80	67.16	97.28	84.57	74.28	94.97	85.28	73.95	95.80	85.62	71.92	90.47	82.87
SD	7.61	6.91	5.13	4.03	4.83	4.49	4.17	3.44	5.59	4.25	4.40	4.17	3.35	3.02

Table S2. The angle of finger joints at maximum extension during grasping and releasing for 22 subjects.

**Angle**	**Thumb**		**Index**			**Middle**			**Ring**			**Little**	
**(°)**	**IP**	**MCP**	**DIP**	**PIP**	**MCP**	**DIP**	**PIP**	**MCP**	**DIP**	**PIP**	**MCP**	**DIP**	**PIP**	**MCP**
S1	11.16	11.15	10.87	27.54	6.77	1.22	25.76	5.78	5.00	21.66	6.78	6.88	19.55	3.65
S2	10.72	18.26	10.49	33.20	16.28	9.01	29.80	11.74	8.63	29.22	12.87	7.96	26.99	11.1
S3	14.11	19.87	5.94	6.29	3.61	2.61	12.71	3.87	4.51	3.65	3.99	3.30	8.30	7.95
S4	15.36	19.55	25.12	42.66	21.54	20.01	38.97	17.77	16.72	35.50	30.44	14.87	28.15	28.03
S5	20.64	13.77	1.02	27.82	13.25	6.25	29.78	13.31	6.68	25.29	6.94	6.4	22.83	1.69
S6	20.93	19.18	3.25	15.95	5.97	4.96	14.71	10.74	3.46	17.09	13.38	6.98	11.45	9.07
S7	8.54	10.56	4.55	14.92	5.14	5.56	10.55	5.04	4.21	8.61	4.52	1.04	13.31	6.03
S8	17.28	9.78	2.54	10.86	7.93	4.07	9.92	4.55	4.71	9.11	8.02	4.93	6.50	3.60
S9	10.18	22.10	2.83	8.40	6.35	7.18	11.58	8.73	4.17	11.18	7.73	1.65	13.68	10.11
S10	19.01	24.89	2.33	15.38	7.35	3.37	18.36	9.51	8.45	17.11	13.48	9.82	16.2	4.37
S11	15.58	18.26	2.41	13.53	8.32	7.13	11.92	9.73	7.15	11.17	8.32	13.06	12.91	3.94
S12	9.41	23.97	6.23	13.07	5.79	5.29	7.28	6.62	5.81	8.72	7.24	1.84	7.75	8.81
S13	18.14	19.69	0.94	15.13	8.84	2.72	10.62	11.82	6.01	12.85	8.06	1.91	11.73	8.78
S14	13.74	20.19	4.02	16.97	7.78	5.13	8.25	8.86	2.82	8.53	5.16	7.51	11.19	9.83
S15	14.02	18.90	0.77	17.85	4.27	7.08	19.60	6.09	6.98	15.18	6.32	1.55	19.53	3.90
S16	19.7	19.52	4.53	17.42	12.73	6.34	18.36	10.89	5.82	17.85	14.71	1.27	21.00	10.28
S17	14.32	23.27	7.14	18.07	14.37	2.91	24.37	15.06	3.95	21.41	14.62	4.75	25.63	10.53
S18	15.45	25.63	2.79	15.47	8.68	4.11	12.83	6.19	5.77	12.27	6.38	4.44	14.23	4.34
S19	8.47	13.66	3.13	16.94	10.43	2.69	18.48	6.41	4.01	17.12	11.71	6.97	10.56	5.52
S20	19.17	26.44	3.21	12.50	5.98	3.67	13.32	5.51	2.82	7.39	7.66	1.96	10.31	7.16
S21	15.62	26.66	1.88	15.31	10.56	1.84	10.29	3.77	1.21	12.94	7.72	1.35	14.51	5.55
S22	19.08	12.84	1.87	12.42	4.68	1.07	10.57	5.28	3.58	9.93	5.91	2.04	12.25	6.66
Mean	15.03	19.01	4.90	17.62	8.94	5.19	16.73	8.51	5.57	15.17	9.63	5.11	15.39	7.77
SD	3.97	5.17	5.29	8.32	4.40	3.93	8.31	3.81	3.10	7.71	5.69	3.91	6.30	5.29

Table S3. Peak velocity of finger joints during flexion for 22 subjects.

Velocity	Thumb		Index			Middle			Ring			Little		
(°/s)	IP	MCP	DIP	PIP	MCP	DIP	PIP	MCP	DIP	PIP	MCP	DIP	PIP	MCP
S1	1051	662	994	1393	1954	1338	1724	1810	1323	1881	1952	1147	1563	1877
S2	818	271	801	1174	974	985	1385	1156	913	1347	1353	1087	1471	1293
S3	755	637	874	1494	1502	1361	1512	1494	1289	1842	1592	1389	1933	1787
S4	949	490	1119	1200	1245	1173	1215	1080	1392	1415	1311	1286	1297	1189
S5	1009	453	982	1254	1476	1221	1707	1495	1200	1871	1675	1352	1897	1905
S6	493	403	1198	1216	995	1014	1535	956	1206	1654	1000	1322	1449	1153
S7	860	545	1010	1338	1563	1323	1588	1603	986	1891	1528	1171	1464	1227
S8	490	290	809	1173	956	974	1126	1053	1115	1220	1131	1129	1206	1181
S9	927	205	844	1443	1610	973	1549	1157	922	1399	1344	1087	1276	1420
S10	636	399	1158	1505	1456	1314	1363	1578	1194	1314	1737	1197	1468	1890
S11	838	525	1396	1566	1811	1659	1938	1724	1418	1596	1811	1437	1497	1675
S12	893	266	939	1487	923	1159	1813	1221	1037	1779	1373	950	1589	802
S13	395	141	711	915	824	916	1062	775	864	1036	943	876	830	760
S14	747	301	862	1448	974	1022	1709	1089	877	1839	1125	1090	1373	973
S15	703	214	694	1156	1216	838	1147	1347	903	1467	1108	958	1449	1532
S16	633	329	1551	1073	1047	1059	895	1168	953	828	951	937	859	1125
S17	750	431	1314	1670	1293	1396	1475	1268	1669	1636	1520	1422	1469	1360
S18	1034	622	1460	1580	1201	1493	1671	1205	1782	1846	1281	1625	1762	1528
S19	644	352	707	1149	857	852	1246	813	780	1289	724	898	1351	908
S20	419	200	666	693	719	643	864	719	713	773	641	616	748	676
S21	621	221	928	1389	973	1020	1424	1152	1119	1427	1282	1389	1361	1053
S22	565	279	1176	1250	1240	1203	1117	1016	1306	1216	1303	1251	1160	1238
Mean	738	375	1009	1298	1219	1134	1412	1222	1135	1480	1304	1164	1385	1298
SD	196	154	258	232	333	241	297	297	277	337	339	234	302	371

Table S4. Peak velocity of finger joints during extension for 22 subjects.

Velocity	Thumb		Index			Middle			Ring			Little		
(°/s)	IP	MCP	DIP	PIP	MCP	DIP	PIP	MCP	DIP	PIP	MCP	DIP	PIP	MCP
S1	949	386	777	1498	1743	1085	1671	1548	1118	1852	1496	1106	1561	1712
S2	715	263	961	1300	1061	1168	1251	1432	950	1031	1484	1089	1112	1325
S3	1026	502	1093	1516	1336	1432	1531	1644	1408	1720	1440	1424	1743	1224
S4	962	610	1199	1448	1508	1121	1207	1463	1527	1601	1479	1478	1399	1636
S5	935	331	1242	1237	1428	1046	1030	1469	1200	1284	1449	829	1187	1492
S6	550	417	1105	1170	1168	1129	1585	1178	1464	1408	1017	1242	1456	1484
S7	848	367	1066	1221	1226	1214	1433	1232	1167	1387	1233	1469	1043	1300
S8	408	257	1187	994	1098	857	1235	1304	1208	1372	1091	1039	1087	1400
S9	827	254	777	1505	1239	963	1548	1039	989	1525	980	894	1382	1429
S10	766	275	1038	1062	1268	1109	1125	1222	1252	1142	1051	910	1184	1499
S11	656	314	983	1145	1337	977	1010	1295	842	1409	1213	1100	1435	1304
S12	927	256	916	1220	1130	1378	1352	1289	1218	1838	1287	1341	2189	1315
S13	300	211	926	837	766	892	1025	709	858	990	950	1177	992	956
S14	764	272	1361	1501	1076	895	1610	1073	818	1326	1061	959	1196	1178
S15	706	312	813	1662	1605	1119	1645	1629	1019	1615	1409	1248	1843	1646
S16	582	261	1416	987	985	806	880	1223	907	823	825	1038	790	1038
S17	808	410	1163	1594	1652	1407	1365	1675	1624	1517	1563	1485	1450	1541
S18	697	326	1329	1400	1300	1409	1656	1255	1578	1914	1107	1615	1669	1342
S19	820	509	1364	1387	1005	1009	1336	1090	957	1556	778	1347	1414	1080
S20	524	203	978	868	815	1065	870	809	849	993	1008	964	808	1033
S21	773	264	1059	1707	991	1017	1463	1200	1064	1701	1165	1395	1726	1376
S22	1008	381	1367	1639	1630	1358	1782	1241	1546	1681	1412	1415	1550	1536
Mean	752	336	1096	1314	1244	1112	1346	1274	1162	1440	1204	1208	1373	1357
SD	192	104	197	259	269	188	272	248	264	303	235	228	344	209

Table S5. The initiation point of flexion of finger joints after time normalization (0~50% for flexion) for 22 subjects.

Position (%)	Thumb		Index			Middle			Ring			Little		
	IP	MCP	DIP	PIP	MCP	DIP	PIP	MCP	DIP	PIP	MCP	DIP	PIP	MCP
S1	9	5	9	2	4	6	2	7	8	1	7	9	3	5
S2	7	2	3	1	4	6	2	3	6	3	4	7	4	5
S3	5	7	7	4	8	12	5	6	5	4	9	11	5	6
S4	6	3	6	4	3	5	6	3	4	5	5	6	3	5
S5	3	6	9	5	6	7	4	5	6	4	5	10	4	7
S6	3	4	10	9	4	12	9	4	9	5	4	15	6	7
S7	4	2	10	4	5	8	6	6	5	6	5	7	6	5
S8	3	1	8	5	3	9	8	4	4	4	3	5	4	7
S9	5	5	12	5	6	8	6	9	8	6	7	11	6	7
S10	3	6	22	18	8	14	6	7	5	6	11	13	8	14
S11	4	12	12	5	6	9	5	9	8	6	7	10	6	5
S12	6	5	14	10	4	15	7	5	10	8	7	19	8	5
S13	10	1	11	5	4	7	4	6	5	4	8	11	3	4
S14	5	4	13	8	4	7	8	5	6	8	4	13	12	6
S15	4	8	9	3	8	11	6	5	13	6	5	12	6	10
S16	1	5	13	9	4	4	8	5	6	5	4	13	5	6
S17	8	5	6	7	2	6	6	2	6	5	3	8	5	4
S18	3	7	9	7	6	12	5	8	7	6	8	12	7	8
S19	3	4	12	6	4	9	7	4	8	7	4	14	4	6
S20	2	3	18	6	7	8	4	6	8	6	8	18	6	5
S21	3	2	14	4	8	9	4	7	9	5	13	16	6	6
S22	3	2	9	6	5	11	3	10	10	4	9	8	5	7
Mean	4.55	4.50	10.73	6.05	5.14	8.86	5.50	5.73	7.09	5.18	6.36	11.27	5.55	6.36
SD	2.30	2.60	4.13	3.48	1.81	2.92	1.92	2.07	2.24	1.59	2.65	3.73	2.04	2.19

Table S6. The maximum velocity point of flexion of finger joints after time normalization (0~50% for flexion) for 22 subjects.

Position	Thumb		Index			Middle			Ring			Little		
(%)	IP	MCP	DIP	PIP	MCP	DIP	PIP	MCP	DIP	PIP	MCP	DIP	PIP	MCP
S1	25	23	26	13	20	31	14	20	31	11	23	24	15	25
S2	26	16	12	13	23	36	15	23	32	15	26	18	16	30
S3	28	24	15	16	27	18	16	22	17	16	26	20	19	28
S4	31	31	21	22	33	19	20	32	19	20	32	19	18	33
S5	23	26	30	16	25	16	15	25	16	13	24	21	13	25
S6	33	34	26	25	32	25	20	26	22	20	30	24	20	34
S7	26	25	35	15	23	25	19	24	21	19	23	30	20	22
S8	29	20	23	17	27	27	21	28	23	17	30	24	19	27
S9	24	18	23	15	25	18	17	27	18	17	28	19	17	32
S10	23	18	30	26	31	36	20	28	21	19	28	35	20	33
S11	21	27	31	19	26	30	15	22	28	20	24	22	15	24
S12	28	29	27	24	16	24	21	30	24	22	32	27	24	34
S13	30	26	37	17	25	35	18	25	20	16	25	21	15	27
S14	28	20	30	18	25	31	18	25	32	19	28	26	20	28
S15	28	18	20	14	21	18	13	19	19	18	26	21	19	29
S16	32	36	34	35	25	23	32	24	16	23	30	37	22	30
S17	33	23	23	20	29	18	18	31	18	19	29	20	18	32
S18	33	32	23	19	31	18	16	29	21	18	30	22	19	30
S19	21	27	17	16	20	19	17	20	29	16	21	22	17	24
S20	37	29	32	20	15	29	18	18	30	20	31	38	18	34
S21	26	27	26	22	29	24	19	28	28	24	31	25	23	31
S22	33	18	18	16	30	17	15	29	17	15	32	19	16	30
Mean	28.09	24.86	25.41	19.00	25.36	24.41	18.05	25.23	22.82	18.05	27.68	24.27	18.32	29.18
SD	4.34	5.64	6.65	5.16	4.89	6.62	3.87	3.98	5.50	3.11	3.33	5.84	2.73	3.61

Table S7. The completion point of flexion of finger joints after time normalization (0~50% for flexion) for 22 subjects.

Position (%)	Thumb		Index			Middle			Ring			Little		
	IP	MCP	DIP	PIP	MCP	DIP	PIP	MCP	DIP	PIP	MCP	DIP	PIP	MCP
S1	31	44	45	39	32	43	32	37	43	33	36	41	35	38
S2	45	45	44	28	43	45	30	43	45	30	41	46	28	42
S3	44	41	41	39	40	43	38	38	43	26	38	42	32	40
S4	44	47	36	41	43	42	38	45	28	33	44	30	30	43
S5	34	46	44	32	40	42	26	40	42	24	38	44	24	38
S6	45	46	44	35	43	46	31	41	44	30	42	44	31	44
S7	44	40	44	39	39	41	37	42	41	36	42	43	35	44
S8	46	45	46	35	39	45	36	43	39	34	44	46	30	43
S9	41	45	46	40	40	40	27	43	44	27	40	43	28	40
S10	47	34	46	41	39	46	41	40	42	38	39	45	42	40
S11	38	44	46	39	40	43	37	36	44	37	35	44	37	36
S12	41	42	45	39	41	45	40	39	42	29	42	43	32	40
S13	47	40	46	36	39	45	35	41	47	33	40	46	36	41
S14	43	45	45	37	38	43	33	40	43	29	41	38	32	40
S15	40	46	44	35	40	45	37	40	46	29	42	42	29	41
S16	44	46	45	45	37	43	45	35	40	44	41	47	42	40
S17	45	44	37	45	39	42	40	42	34	39	45	42	38	44
S18	44	46	44	41	42	44	29	42	32	31	44	43	33	45
S19	36	42	44	36	41	44	28	40	45	25	38	39	29	42
S20	46	45	42	41	45	46	37	43	47	40	42	48	38	44
S21	46	46	46	35	44	44	30	43	44	31	43	46	36	42
S22	44	45	46	44	41	42	31	44	45	28	43	45	30	40
Mean	42.50	43.82	43.91	38.27	40.23	43.59	34.45	40.77	41.82	32.09	40.91	43.05	33.05	41.23
SD	4.30	2.99	2.74	4.13	2.72	1.68	5.07	2.60	4.81	5.20	2.67	3.81	4.63	2.31

Table S8. The initiation point of extension of finger joints after time normalization (50~100% for extension) for 22 subjects.

Position (%)	Thumb		Index			Middle			Ring			Little		
	IP	MCP	DIP	PIP	MCP	DIP	PIP	MCP	DIP	PIP	MCP	DIP	PIP	MCP
S1	59	53	52	65	64	54	76	60	55	75	57	53	74	58
S2	53	53	56	70	56	60	71	56	57	68	57	61	68	58
S3	58	54	58	57	59	54	62	66	54	68	61	55	65	55
S4	55	53	60	59	56	55	63	60	58	58	57	64	59	60
S5	54	53	56	65	58	55	69	60	62	68	56	54	70	57
S6	54	54	56	65	56	55	67	58	55	67	56	57	67	55
S7	59	51	53	57	56	53	64	58	58	60	54	59	60	54
S8	54	51	53	60	55	53	66	60	58	60	53	53	60	54
S9	55	54	53	59	58	56	69	57	54	72	58	54	70	59
S10	51	53	51	58	57	53	63	59	57	61	53	58	59	54
S11	56	53	54	61	58	57	64	57	54	69	60	69	67	55
S12	61	54	57	66	56	56	69	57	55	67	56	55	66	57
S13	51	53	52	59	56	53	66	59	53	62	54	63	59	53
S14	56	54	53	60	57	53	70	60	57	65	54	56	63	55
S15	54	54	53	62	57	54	65	61	53	65	57	58	64	56
S16	56	51	53	53	58	58	53	60	58	54	57	60	56	56
S17	55	54	52	58	60	55	63	61	64	60	58	58	63	57
S18	53	55	54	60	58	57	68	58	64	67	55	61	63	56
S19	55	54	56	58	57	53	65	58	53	66	59	52	63	58
S20	52	52	55	56	53	53	57	53	53	58	54	54	61	53
S21	55	51	54	66	57	54	66	56	55	67	57	53	64	59
S22	54	57	57	58	63	54	64	59	55	59	56	55	59	56
Mean	55.00	53.23	54.45	60.55	57.50	54.77	65.45	58.77	56.45	64.36	56.32	57.36	63.64	56.14
SD	2.53	1.45	2.28	4.07	2.43	1.90	4.75	2.51	3.32	5.14	2.15	4.29	4.46	1.98

Table S9. The maximum velocity point of extension of finger joints after time normalization (50~100% for extension) for 22 subjects.

Position (%)	Thumb		Index			Middle			Ring			Little		
	IP	MCP	DIP	PIP	MCP	DIP	PIP	MCP	DIP	PIP	MCP	DIP	PIP	MCP
S1	73	85	83	88	81	71	88	79	69	88	78	81	90	78
S2	71	82	77	86	83	73	86	82	71	84	73	84	88	74
S3	74	78	75	76	75	73	82	76	78	78	77	78	80	75
S4	71	67	81	82	80	83	82	81	84	75	82	82	83	80
S5	72	84	75	81	80	75	84	83	72	85	80	82	88	78
S6	70	65	75	82	71	76	82	78	76	82	70	77	85	69
S7	74	81	67	74	73	75	76	76	71	72	69	66	77	67
S8	69	55	66	77	72	73	77	76	70	72	67	67	74	69
S9	78	65	71	77	78	77	79	80	79	81	82	75	83	82
S10	61	74	67	71	70	67	75	73	75	74	72	66	76	71
S11	67	78	81	82	79	78	79	80	80	81	82	75	81	82
S12	71	79	76	79	70	79	80	73	74	76	75	75	77	80
S13	58	62	62	70	67	62	76	73	72	75	68	70	78	67
S14	71	74	69	75	82	77	80	78	64	77	71	70	81	71
S15	78	77	74	75	77	74	78	79	76	80	81	73	75	79
S16	77	61	73	69	75	77	73	78	70	81	73	75	77	73
S17	76	70	75	82	82	81	81	84	81	82	82	79	86	85
S18	68	82	79	81	78	79	84	78	81	82	80	80	82	73
S19	68	67	69	71	72	73	78	75	76	75	74	74	77	75
S20	60	67	64	68	65	65	67	65	65	70	64	64	74	65
S21	78	67	67	80	72	64	78	79	76	80	82	71	79	80
S22	67	73	70	74	75	75	76	78	66	75	68	76	77	74
Mean	70.55	72.41	72.55	77.27	75.32	73.95	79.14	77.45	73.91	78.41	75.00	74.55	80.36	74.86
SD	5.65	8.33	5.80	5.51	5.09	5.42	4.59	4.11	5.43	4.67	5.86	5.70	4.75	5.56

Table S10. The completion point of extension of finger joints after time normalization (50~100% for extension) for 22 subjects.

Position (%)	Thumb		Index			Middle			Ring			Little		
	IP	MCP	DIP	PIP	MCP	DIP	PIP	MCP	DIP	PIP	MCP	DIP	PIP	MCP
S1	95	96	96	99	95	94	99	97	93	98	97	94	99	90
S2	96	98	94	97	94	96	98	93	96	99	92	95	98	93
S3	88	95	86	91	94	82	89	95	91	93	91	85	91	95
S4	90	96	95	97	95	96	97	93	96	96	96	95	96	94
S5	89	98	88	95	94	91	96	92	90	97	93	94	97	89
S6	95	94	89	94	87	88	93	91	89	93	90	86	94	89
S7	92	96	82	90	91	90	92	92	92	92	83	92	93	81
S8	94	95	85	93	83	85	94	87	88	93	84	88	93	81
S9	90	94	80	91	89	87	95	89	88	94	92	88	94	91
S10	91	97	83	92	84	79	93	87	92	92	89	90	92	79
S11	92	96	89	97	90	95	96	91	90	94	92	90	92	91
S12	90	94	85	90	92	86	94	90	86	88	94	82	85	93
S13	96	88	84	91	90	86	92	92	87	91	88	80	91	85
S14	92	95	78	90	92	91	93	92	91	91	86	92	92	88
S15	89	94	85	90	88	82	89	91	85	90	91	83	84	92
S16	97	95	91	92	93	91	90	90	89	93	93	90	94	90
S17	93	93	90	95	93	94	97	95	93	94	94	97	97	93
S18	93	98	87	97	91	89	95	92	92	94	93	85	93	91
S19	86	93	74	86	92	88	90	92	82	84	94	79	89	92
S20	95	94	78	90	92	86	92	90	89	92	93	81	93	92
S21	97	92	86	92	92	88	92	89	88	92	92	83	89	93
S22	88	95	81	90	92	80	90	92	82	90	93	87	90	92
Mean	92.18	94.82	85.73	92.68	91.05	88.36	93.45	91.45	89.5	92.73	91.36	88.00	92.55	89.73
SD	3.19	2.24	5.66	3.27	3.21	4.96	2.92	2.40	3.73	3.27	3.53	5.34	3.76	4.41

Table S11. The initiation point and completion point of flexion of fingers after time normalization (0~50% for flexion) for 22 subjects.

Position (%)	Initiation point					Completion point				
	Thumb	Index	Middle	Ring	Little	Thumb	Index	Middle	Ring	Little
S1	5	2	2	1	3	44	45	43	43	41
S2	2	1	2	3	4	45	44	45	45	46
S3	5	4	5	4	5	44	41	43	43	42
S4	3	3	3	4	3	47	43	45	44	43
S5	3	5	4	4	4	46	44	42	42	44
S6	3	4	4	4	6	46	44	46	44	44
S7	2	4	6	5	5	44	44	42	42	44
S8	1	3	4	3	4	46	46	45	44	46
S9	5	5	6	6	6	45	46	43	44	43
S10	3	8	6	5	8	47	46	46	42	45
S11	4	5	5	6	5	44	46	43	44	44
S12	5	4	5	7	5	42	45	45	42	43
S13	1	4	4	4	3	47	46	45	47	46
S14	4	4	5	4	6	45	45	43	43	40
S15	4	3	5	5	6	46	44	45	46	42
S16	1	4	4	4	5	46	45	45	44	47
S17	5	2	2	3	4	45	45	42	45	44
S18	3	6	5	6	7	46	44	44	44	45
S19	3	4	4	4	4	42	44	44	45	42
S20	2	6	4	6	5	46	45	46	47	48
S21	2	4	4	5	6	46	46	44	44	46
S22	2	5	3	4	5	45	46	44	45	45
Mean	3.09	4.09	4.18	4.41	4.95	45.18	44.73	44.09	44.05	44.09
SD	1.38	1.51	1.22	1.33	1.29	1.40	1.24	1.31	1.46	2.00

Table S12. The initiation point and completion point of extension of fingers after time normalization (50~100% for extension) for 22 subjects.

Position (%)	Initiation point					Completion point				
	Thumb	Index	Middle	Ring	Little	Thumb	Index	Middle	Ring	Little
S1	53	52	54	55	53	96	99	99	98	99
S2	53	56	56	57	58	98	97	98	99	98
S3	54	57	54	54	55	95	94	95	93	95
S4	53	56	55	57	59	96	97	97	96	96
S5	53	56	55	56	54	98	95	96	97	97
S6	54	56	55	55	55	95	94	93	93	94
S7	51	53	53	54	54	96	91	92	92	93
S8	51	53	53	53	53	95	93	94	93	93
S9	54	53	56	54	54	94	91	95	94	94
S10	51	51	53	53	54	97	92	93	92	92
S11	53	54	57	54	55	96	97	96	94	92
S12	54	56	56	55	55	94	92	94	94	93
S13	51	52	53	53	53	96	91	92	91	91
S14	54	53	53	54	55	95	92	93	91	92
S15	54	53	54	53	56	94	90	91	91	92
S16	51	53	53	54	56	97	93	91	93	94
S17	54	52	55	58	57	93	95	97	94	97
S18	53	54	57	55	56	98	97	95	94	93
S19	54	56	53	53	52	93	92	92	94	92
S20	52	53	53	53	53	95	92	92	93	93
S21	51	54	54	55	53	97	92	92	92	93
S22	54	57	54	55	55	95	92	92	93	92
Mean	52.82	54.09	54.36	54.55	54.77	95.59	93.55	94.05	93.68	93.86
SD	1.26	1.82	1.36	1.44	1.74	1.50	2.50	2.34	2.15	2.21
